# Logic Gate Operation by DNA Translocation through Biological Nanopores

**DOI:** 10.1371/journal.pone.0149667

**Published:** 2016-02-18

**Authors:** Hiroki Yasuga, Ryuji Kawano, Masahiro Takinoue, Yutaro Tsuji, Toshihisa Osaki, Koki Kamiya, Norihisa Miki, Shoji Takeuchi

**Affiliations:** 1 Artificial Cell Membrane Systems Group, Kanagawa Academy of Science and Technology, Kawasaki, Japan; 2 Department of Mechanical Engineering, Keio University, Yokohama, Japan; 3 Interdisciplinary Graduate School of Science and Engineering, Tokyo Institute of Technology, Yokohama, Japan; 4 Institute of Industrial Science, The University of Tokyo, Tokyo, Japan; 5 Department of Biotechnology and Life Science, Tokyo University of Agriculture and Technology, Tokyo, Japan; University of Iowa, UNITED STATES

## Abstract

Logical operations using biological molecules, such as DNA computing or programmable diagnosis using DNA, have recently received attention. Challenges remain with respect to the development of such systems, including label-free output detection and the rapidity of operation. Here, we propose integration of biological nanopores with DNA molecules for development of a logical operating system. We configured outputs “1” and “0” as single-stranded DNA (ssDNA) that is or is not translocated through a nanopore; unlabeled DNA was detected electrically. A negative-AND (NAND) operation was successfully conducted within approximately 10 min, which is rapid compared with previous studies using unlabeled DNA. In addition, this operation was executed in a four-droplet network. DNA molecules and associated information were transferred among droplets via biological nanopores. This system would facilitate linking of molecules and electronic interfaces. Thus, it could be applied to molecular robotics, genetic engineering, and even medical diagnosis and treatment.

## Introduction

DNA/RNA computing has recently received attention as a bioinspired method capable of executing massively parallel computing [1, 2] or large-scale molecular logic circuits [3–5] based on hybridization reactions [1–5], enzymatic reactions [2, 5], and strand displacement reactions [4]. DNA/RNA computing has recently been implemented in extensive applications, such as imitation of neural networks [6], cellular drug delivery [7], and programmable diagnosis [8, 9]. The results of conventional methods of DNA/RNA computing are output as DNA and RNA molecules. The molecules containing the output information, such as diagnostic results, must be processed as human-recognizable information, e.g. electrical binary data. However, in conventional systems, multistep procedures such as PCR, gel electrophoresis, and fluorescent labeling are required for the readout of output signals, which delays completion of the readout. Moreover, the molecular outputs cannot be directly connected to versatile electronic devices.

To address these issues, we here propose a system for electrical detection of unlabeled DNA as the output of a logic operation using an α-hemolysin (αHL) nanopore, for constructing a simple logic gate. αHL nanopores have a diameter of 1.4 nm, which matches the size of DNA molecules (~1 nm in diameter for single strands); given their size, αHL nanopores have been used to detect or sequence single-stranded DNA (ssDNA) [10, 11]. Translocation of DNA through the nanopore can be detected by the blocking of the channel current signal, with a high signal-to-noise ratio [12, 13]. In this study, we demonstrate a label-free and electrically rapid negative-AND (NAND) operation applied to a DNA and nanopore system in droplet network.

**Fig 1:**
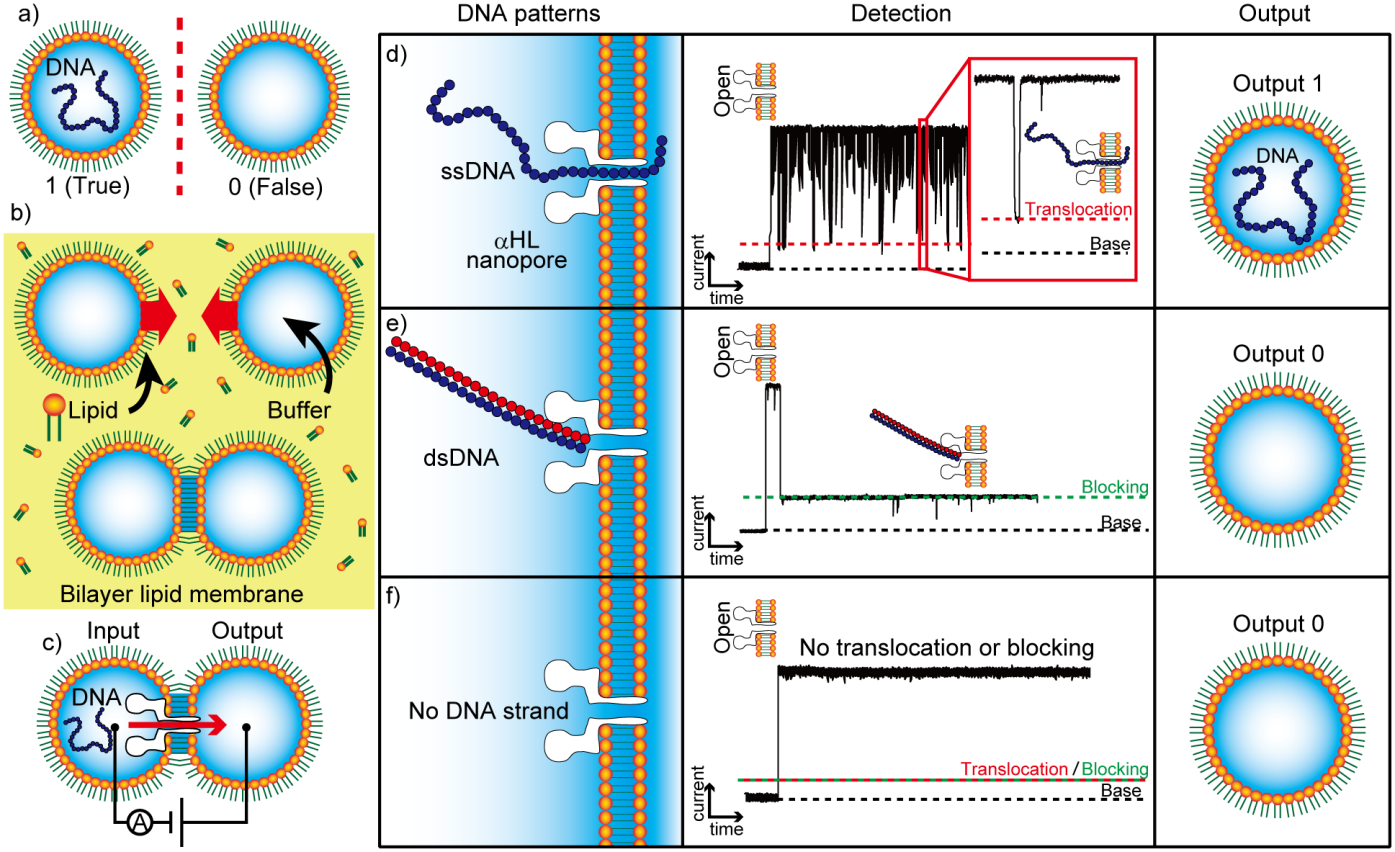
Droplet contact method (DCM) and the binary system based on DNA blocking and translocation. (a) Definition of the binary system based on the presence of DNA in an aqueous droplet coated with a lipid monolayer. (b) A schematic view of the bilayer lipid membrane (BLM) formed via DCM. (c) The system for DNA transfer among droplets by DNA translocation through αHL nanopores. (d–f) Electrical detection of DNA translocation and determination of the presence of DNA constructs in the droplet. (d) Output 1 (translocation): A single-stranded DNA (ssDNA) is translocated through an αHL nanopore with a short current blockade. (e) Output 0 (blocking): A double-stranded DNA (dsDNA) is not translocated through the αHL nanopore owing to its larger diameter, inducing a long current blockade. (f) Output 0 (No DNA strand): No DNA strand is present for translocation, and thus no current blockade is generated.

The binary states of our nanopore system are shown in Fig 1a; true is defined as the state in which DNA strands are present in a lipid-coated droplet and false is defined as the absence of DNA. A bilayer lipid membrane (BLM) is formed by contact between lipid-coated droplets (Fig 1b), known as the droplet contact method (DCM) [14–17]. The αHL nanopore is reconstituted in the BLM, and DNA strands are driven towards the nanopore following a positive voltage gradient, as shown in Fig 1c. The true state can be obtained when ssDNA in the input droplet is translocated through the αHL nanopore and into the output droplet. At that time, we can detect the output signal electrically because ssDNA inhibits ionic currents from travelling through the pore during translocation [18], as shown in Fig 1d. Each translocation event produces a peak-like current blockade in the channel current recordings. The false state indicates the absence of DNA in the output droplet, but electrical signals are produced in two different cases. One is the blocking of the αHL nanopore by double-stranded DNA (dsDNA) (Fig 1e). dsDNA (diameter ~2 nm, which is greater than the 1.4 nm diameter of the αHL pore) blocks the αHL nanopore, which induces a long current blockade (>1 sec). In the other case, no DNA strand moves to the pore (Fig 1f). In our binary system, outputs can be estimated from signals detected by electrical observations. The method enables rapid readouts, owing to the rapidity of DNA translocation.

## Results and Discussion

### Principal of NAND operation system using nanopores

**Fig 2:**
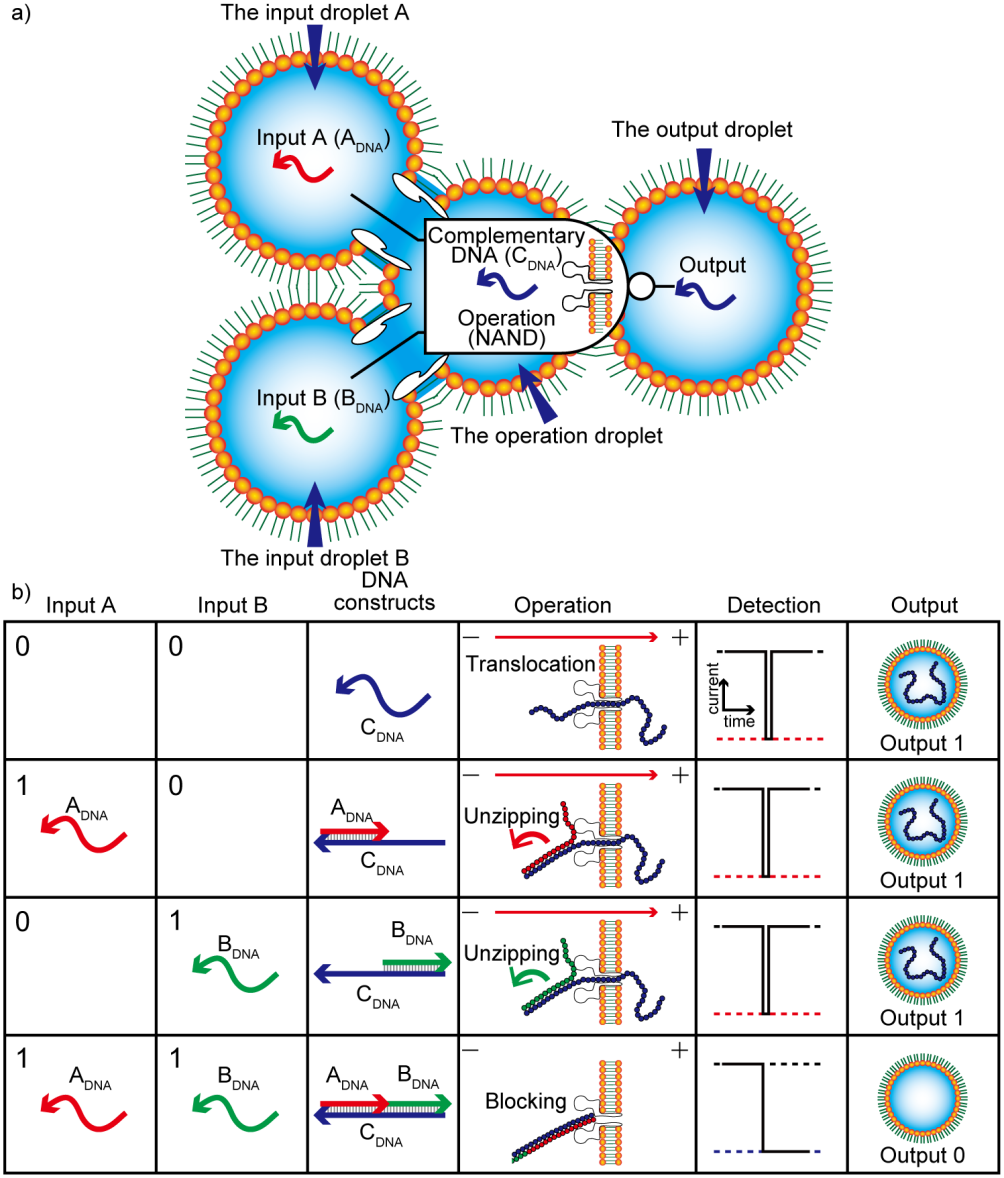
Negative-AND (NAND) operation system based on DNA translocation. (a) NAND logic gate concept based on αHL nanopores and three types of DNA strands, A_DNA_, B_DNA_, and complementary DNA (C_DNA_) in a droplet network. (b) A schematic table of NAND operations with the DNA structures and nanopore result for each input.

To demonstrate the general applicability of logic gates based on DNA translocation, we attempted to construct a NAND gate, because NAND operations can be converted into all other logical operations (AND, OR, NOT, etc.). The NAND logic gate developed in this study is shown in Fig 2a. Three types of ssDNA are prepared for this operation. A_DNA_ and B_DNA_ are used as Input A and Input B, respectively. In addition, a complementary DNA (C_DNA_) is applied to determine the output binary: The presence and absence of C_DNA_ in the output droplet determine output 1 and 0, respectively. The C_DNA_ is twice as long as the input DNAs and hybridized to both A_DNA_ and B_DNA_ at different termini. The three DNA types (A_DNA_, B_DNA_, and C_DNA_) form a complete duplex structure because the length of A_DNA_ + B_DNA_ is equal to that of the C_DNA_. The NAND operation can be described as follows and is shown in Fig 2b.

Input (A→0, B→0): When no input DNA strands are present, C_DNA_ strands are present in the operation droplet. Along with the voltage potential over αHL, C_DNA_ strands are translocated through the αHL nanopore, resulting in a peak-like current blockade, and stored in the output droplet, i.e., output 1.

Input (A→1, B→0): The A_DNA_ and C_DNA_ strands are present in the operation droplet after the A_DNA_ is translocated from input droplet A. The A_DNA_ strands hybridize with the C_DNA_, but a single-stranded region remains. After the voltage is applied, the single-stranded region enters the nanopore, and the duplex region is unzipped at the vestibule of the nanopore, and finally the A_DNA_-C_DNA_ duplex is decomposed and translocated through the pore [19]. During translocation, we observe a peak-like current blockade, and the C_DNA_ is stored in the output droplet, generating an output of 1.

Input (A→0, B→1): The B_DNA_ and C_DNA_ strands are present in the operation droplet after the B_DNA_ is translocated from input droplet B. Via the same mechanism described for input (1, 0) above, C_DNA_ is translocated through the αHL nanopore, producing output 1.

Input (A→1, B→1): A_DNA_, B_DNA_, and C_DNA_ strands are present in the operation droplet after both A_DNA_ and B_DNA_ are translocated from input droplets A and B, respectively. They hybridize to form dsDNA without single-stranded regions. When a voltage is applied, the dsDNA will enter the vestibule of the αHL nanopore. However, the dsDNA cannot be unzipped since it does not contain a single-stranded region; it therefore remains in the vestibule because it is larger than the constriction of the nanopore. This blocking event produces a long current blockade. In this case, no C_DNA_ is stored in the output droplet, i.e., output 0.

### Design of DNA for the NAND gate using a thermodynamic simulation

To appropriately execute the NAND operation, DNA must be translocated through an αHL nanopore for inputs (0, 0), (0, 1), and (1, 0), but must not be translocated when the input is (1, 1). For the input (0, 0), we prepared a C_DNA_ that would pass through the pore even without DNA inputs. For inputs (1, 0) and (0, 1), the C_DNA_ contained a partially double-stranded region; the region was composed of C_DNA_ hybridized with either A_DNA_ or B_DNA_. For these DNA molecules to pass through the αHL, we took advantage of the “unzipping” behavior of dsDNA. The mechanism of unzipping at the αHL nanopore operates as follows. First, the ssDNA region enters the vestibule of the αHL (1.4 nm in diameter) but the dsDNA region cannot pass through the nanopore (Fig 2b) [19]. Unzipping of the double-stranded region then occurs when the free energy of hybridization is lower than that of the external force resulting from the applied voltage (Fig 2b). Consequently, the C_DNA_ passes through the pore after unzipping. However, for input (1, 1), A_DNA_ + B_DNA_ are completely hybridized to the C_DNA_. This fully dsDNA cannot pass through the nanopore.

Under our experimental conditions (1 M KCl and 120 mV voltage application), 332 kJ mol^-1^ was estimated as the upper limit of the free energy for unzipping [19]. For free energies above the limit, unzipping would not occur within 1 sec. Therefore, the free energy of the hybridization of C_DNA_ with A_DNA_ and B_DNA_ were carefully calculated, so as not to exceed the limit, by thermodynamic simulation using Nupack [20] (see Tables 1 and 2 and DNA design and free energy calculation in Materials and Methods). The free energy of hybridization of guanine (G) and cytosine (C) is higher than that of adenine (A) and thymine (T). It is therefore necessary to determine a number of poly-G and -C bases with a free energy close to the upper limit, and to select a sequence with fewer bases accordingly. Based on the simulation, the free energy of a sequence composed of approximately 40 poly-GC nucleotides (341.1 kJ) exceeds the limit. Therefore, we selected a length of 20 bases for the A_DNA_ and B_DNA_ sequences. The length of the C_DNA_ was set at 40 bases. To avoid formation of unexpected internal secondary structures, the A_DNA_ and B_DNA_ strands were composed of T and C residues, whereas the C_DNA_ strands were composed of A and G residues. The A_DNA_ and B_DNA_ strands were intended to hybridize to the 3′ and 5′ termini of the C_DNA_ strand, respectively, owing to the different base pairing. For input (1, 1), duplex DNA is necessary at both termini to prevent translocation. The DNA design described above satisfies these requirements. Although dsDNA composed of A_DNA_, B_DNA_, and C_DNA_ does not exceed the upper limit (Table 2), unzipping does not occur because no single-stranded region is present to be inserted into the constricted region. The hybridization of the DNA strands was confirmed by gel electrophoresis, as shown S1 Fig (see also Gel Electrophoresis in S1 Text).

**Table 1:** Nucleotide sequences of A_DNA_, B_DNA_, and complementary DNA (C_DNA_).

DNA	Base sequence
**Input A**_**DNA**_	5′-TTTTCCCTTTCCTTTCTTTC-3′(20-mer)
**Input B**_**DNA**_	5′-CCTTCCTTCTTCCCTCCTCT-3′(20-mer)
**Complementary DNA (C**_**DNA**_**)**	5′-AGAGGAGGGAAGAAGGAAGGGAAAGAAAGGAAAGGGAAAA-3′(40mer)

**Table 2:** Free energy of the secondary structures of DNA constructs.

DNA constructs	Free Energy [kJ/mol]
**A**_**DNA**_ **+ C**_**DNA**_	-134.5
**B**_**DNA**_ **+ C**_**DNA**_	-123.6
**A**_**DNA**_ **+ B**_**DNA**_ **+ C**_**DNA**_	-252.8

### Verification of the NAND operation in the two-droplet system

**Fig 3:**
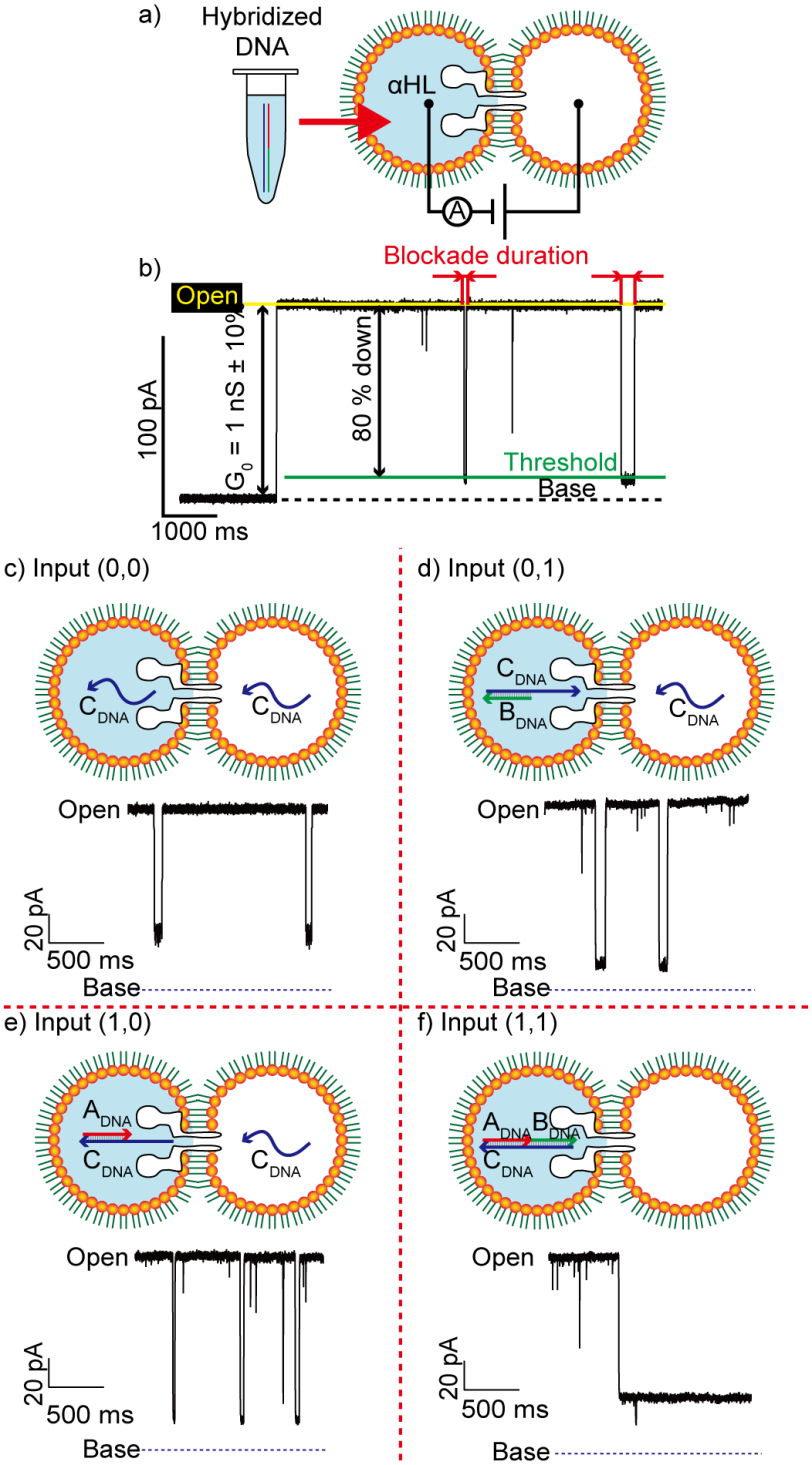
An overview of the experiment performed to confirm that the desired outputs were obtained. (a) Schematic view of the experiment. DNA constructs, hybridized in advance, were injected into a droplet. (b) Typical method for channel current signal analysis. (c–f) A schematic view of translocation of the DNA strands present in each input and the current blockade signals produced.

To confirm the performance of the NAND operation using the designed DNA, an experiment was conducted using a double-well chip (DWC) [17]. We prepared four solution types corresponding to all types of inputs. The solution for input (0, 0) included C_DNA_, that for input (1, 0) included A_DNA_ and C_DNA_, that for input (0, 1) included B_DNA_ and C_DNA_, and that for input (1, 1) included A_DNA_, B_DNA_, and C_DNA_. As shown in Fig 3a, the channel current was measured while the voltage was applied across the two droplets. αHL reconstitution was recognized as a rise in current with a conductance of 1 nS (±10%). The conductance was set to G_0_. The threshold for DNA blocking was set to 80% of G_0_. The blockade duration was defined as the time elapsed during the current drop from the open level. Typical channel current and analysis methods are shown in Fig 3b. As shown in Fig 3c–3f, short current-blocking events (< 1 sec) were dominantly observed for inputs (0, 0), (0, 1), and (1, 0), whereas long events (i.e., those lasting several seconds) were observed for input (1, 1). For determination of whether the output was “0” or “1,” we empirically formulated a computing protocol, as described in S1 Scheme. The results showing the NAND operation outputs with respect to the protocol are presented in S2 Fig. We defined the calculation time as the time between the first and the tenth blocking events. The calculation time for each operation was 54.0 s ± 19.7 s (input (0, 0)), 20.3 s ± 10.9 s (input (0, 1)), 20.2 s ± 7.5 s (input (1, 0)), and 117.7 s ± 42.1 s (input (1, 1)), respectively. The values obtained appeared to differ, but when a *t*-test was applied to the calculation times, no significant difference in input patterns was detected (p > 0.05, double-sided). The calculation time required for our system is short compared to that required for conventional logic gates using DNA, which require at least several minutes [21].

### NAND operation in a four-droplet network

**Fig 4:**
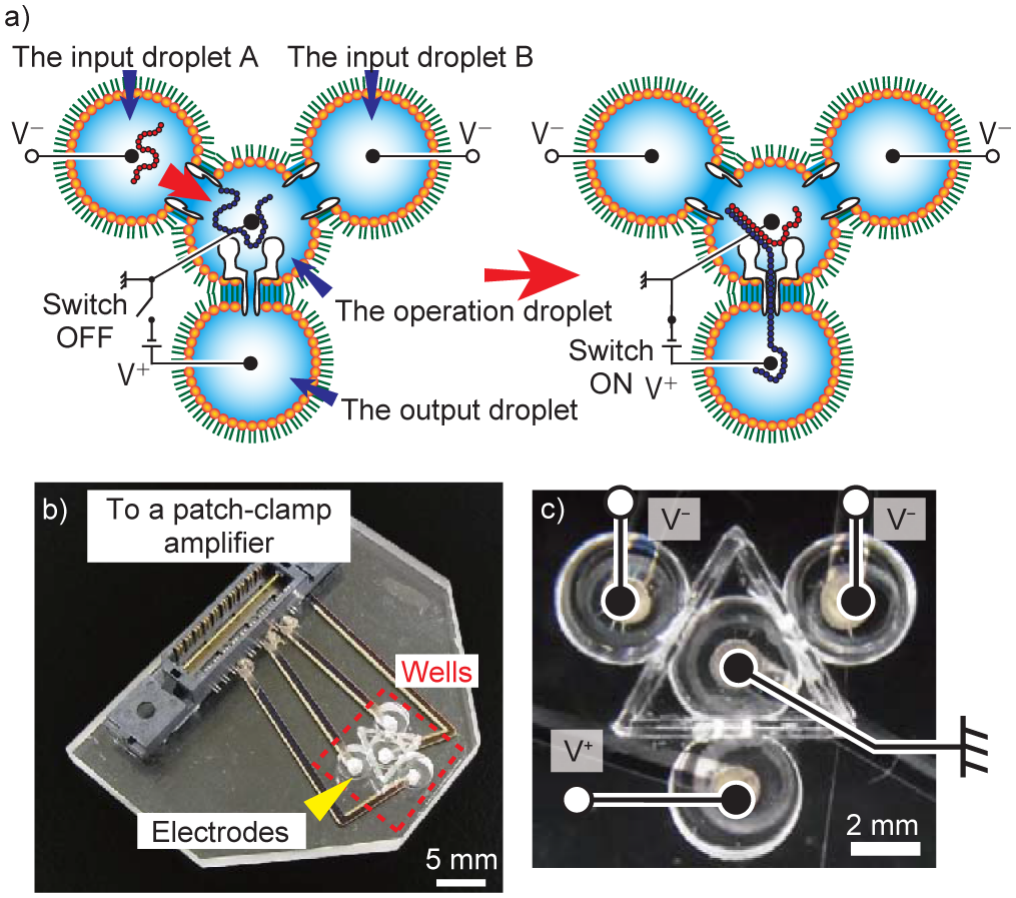
A schematic view of the four-well chip, the device used for the four-droplet network. (a) A four-droplet network for Negative-AND operation. Input (1, 0) was selected as an example. The input DNA strands were injected into the input droplets and the C_DNA_ strands were prepared in the operation droplet. (b) An overall view of the 4WC. The electrodes in the wells were connected to a patch-clamp amplifier. (c) The wiring to the four wells. The electrodes were embedded on the bottom of the wells.

Using the same computing protocol as described in the previous section, we expanded this system into a four-droplet network (Fig 4a). Expansion enables application of a voltage to trigger DNA translocation, i.e. input, operation, and output. This feature is expected to enhance the electrical compatibility of general DNA computing and, moreover, is beneficial for sequential or parallel connection of NAND logic gates. Droplets are connected via biological nanopores [22]. Input droplets A and B containing A_DNA_ and B_DNA_ strands, respectively, were prepared. C_DNA_ was prepared for all operation droplets (Fig 4a). Then, voltages were applied between the input droplets and the operation droplet (Fig 4a left). We selected streptolysin O (SLO) instead of αHL nanopores for the interface between the input and operation droplets because αHL pores are unsuitable for a high rate of DNA translocation due to their small pore size (see Verification of DNA translocation in S1 Text). Voltages were applied between the operation droplet and the output droplet (Fig 4a, right). As the substrate for the network, a four-well chip (4WC) was fabricated (Fig 4b and 4c). The procedure used for construction of the four-droplet network used in the NAND operation is shown in S3 Fig. Initially, *n*-decane containing phospholipids and cholesterol was added to all four wells. Next, aqueous droplets containing the appropriate membrane proteins were respectively injected into the wells. Successive BLM formation and membrane protein reconstitution occurred spontaneously.

**Fig 5:**
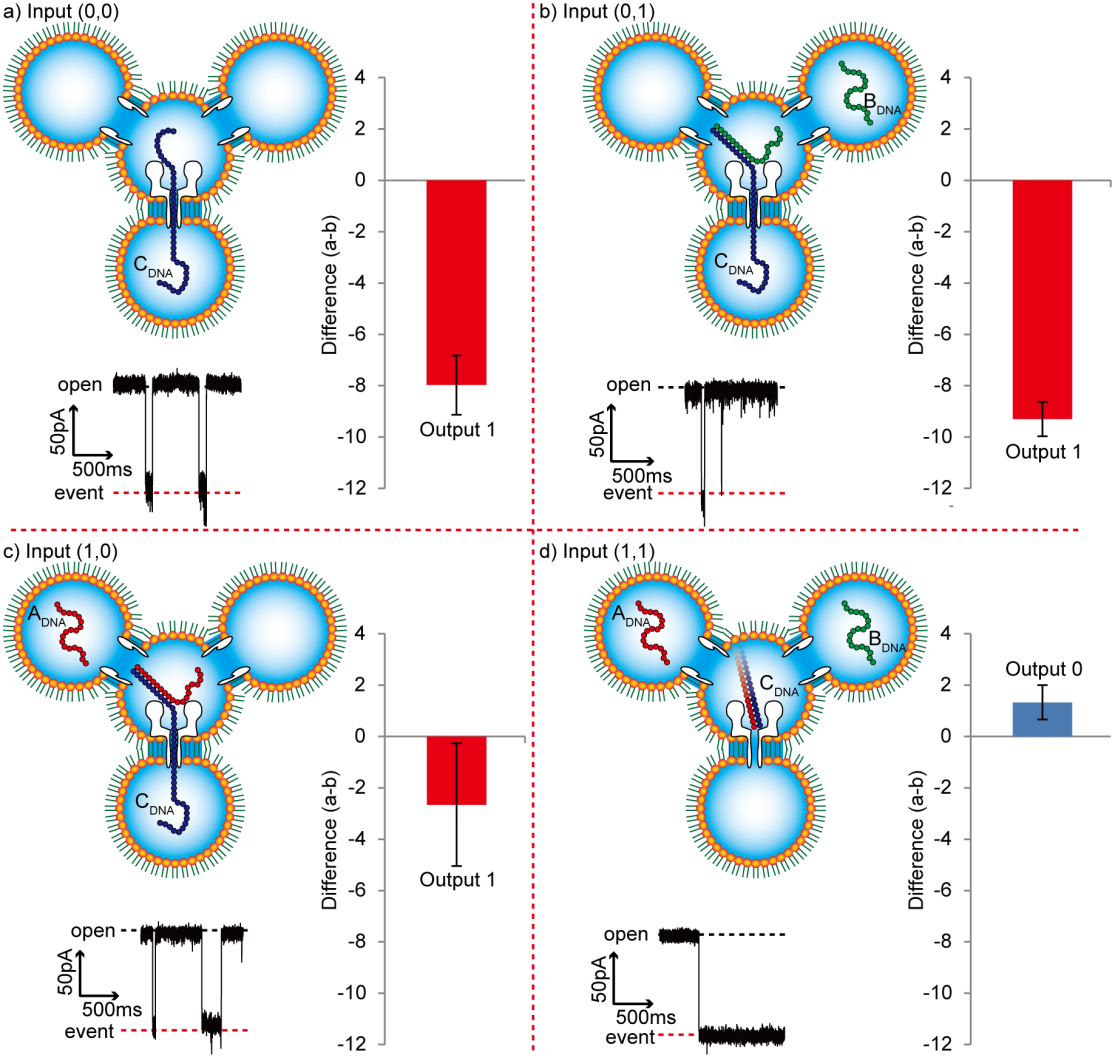
Outputs of the Negative-AND operation in a four-droplet network. The operation was performed as follows: Collect 10 DNA translocation or blocking events for a single calculation. Measure duration of each blocking event. Differentiate the events into two groups, those greater than 1 s (number of events = a) and those not greater than 1 s (number of events = b). Calculate a–b, and determine the output as: Output 1: a–b < 0 or Output 0: a–b > 0. Standard errors were determined using the output from three operations. (a) input (0, 0): a–b = –8.0 ± 1.2, output = 1. (b) input (0, 1): a–b = –9.3 ± 0.7, output = 1. (c) input (1, 0): a–b = –2.7 ± 2.4, output = 1. (d) input (1, 1): a–b = 1.3 ± 0.7, output = 0.

We executed the NAND operation using the four-droplet network with the αHL and SLO nanopores. After the four-droplet network was prepared, with the input DNA in the input droplets, the operation was initiated by applying a voltage between the input and operation droplets for 10 min. The input DNA strands were expected to be translocated into the operation droplet. Next, a voltage was applied between the operation and the output droplet, where DNA translocation was electrically observed. As shown in the current-time trace in Fig 5, blocking events of short duration were dominantly observed for inputs (0, 0), (0, 1), and (1, 0), whereas long events were dominantly observed for input (1, 1). These results are similar to those observed in the experiment using a DWC described above. As shown in the bar graph in Fig 5, the NAND operation occurred and the total operation time was approximately 10 minutes, including the voltage application and the calculation time. This result demonstrates the successful application of biological nanopores in a droplet network for a logical operating system.

According to previous reports, fluorescent-based DNA logic gates generally require minutes to days [21]. In our NAND operation using a four-droplet network, output results can be obtained within approximately 10 min, owing to the electrical detection. However, output accuracy remains a challenge. To guarantee output accuracy, we evaluated ten DNA blocking events in the above mentioned experiments; it was impossible to determine the output from a single DNA blocking event because there was a risk of the opposite output due to probabilistic fluctuation of the blocking time. For more accurate computation, the DNA structure could be further optimized. An optimal molecule might possess a circular region, for rigid structural blocking of the αHL nanopore. That would further accelerate the operation and might also improve accuracy.

## Conclusion

In summary, we developed a NAND operation system on a four-droplet network using three types of DNA. Here, output 1 was determined by ssDNA translocation through an αHL nanopore, whereas output 0 was determined by the absence of DNA translocation. Following the operation, which finishes approximately in 10 min, NAND outputs were successfully detected using the protocol described here. This result is the first example of a DNA logic operation using electrical detection without fluorescent labels to our knowledge.

The principle could be expanded to other programmable operations; for example, small molecules or proteins could be used as inputs if using DNA aptamers [23]. Moreover, our electrical DNA computing system could be adapted for use as an interface between chemical molecules and electronic systems. It should be noted that the output molecules are directly transduced to electrical signals. DNA computing has recently been applied to molecular robotics [24], genetic engineering [9], and even medical diagnosis and treatment [8,9]. Compatibility with electronics is imperative for future practical applications.

## Materials and Methods

### Chemicals

In all experiments, egg yolk phosphatidylcholine (EggPC, Avanti Polar Lipids, Alabaster, AL, USA), cholesterol (chol, Avanti Polar Lipids), and *n*-decane (Sigma-Aldrich, St. Louis, MO, USA) were used. All aqueous solutions were prepared with ultrapure water from a Milli-Q system (Millipore, Billerica, MA, USA). KCl, K_2_HPO_4_, KH_2_PO_4_, and EDTA were purchased from Wako Pure Chemical Industries, Ltd. (Osaka, Japan). A buffered electrolyte solution (1.0 M KCl, 2 mM KH_2_PO_4_, 8 mM K_2_HPO_4_, and 10 mM EDTA adjusted to a pH of 7.4) was prepared. Wild-type alpha-hemolysin (αHL) (Sigma-Aldrich) was used as the monomer polypeptide. αHL was dissolved at a concentration of 1.0 mg mL^-1^ and diluted to the designated concentration using a buffered electrolyte solution. αHL, an exotoxin secreted by the bacterium *Staphylococcus aureus*, is a polypeptide consisting of 293 amino acids [25]. The monomers spontaneously assembled in a BLM and formed oligomeric cylindrical pores with an internal diameter of approximately 1.4 nm [26]. αHL was always diluted to 30 nM for use in experiments. Streptolysin O (SLO, 1.0 mg mL^-1^) (Bio Academia, Ltd., Osaka, Japan) was diluted to the designated concentration using the buffered electrolyte solution. SLO is a membrane-damaging toxic protein produced by group A streptococci that forms arc-shaped or ring-shaped structures on the BLM after binding to cholesterol. The resulting oligomeric cylindrical pores have an internal diameter of up to approximately 25 nm [27]. SLO was diluted to 10 μg mL^-1^ for use in all experiments. Three types of DNA, A_DNA_, B_DNA_, and C_DNA_, as shown in Table 1, were purchased from BEX (Tokyo, Japan). In experiments using a two-droplet system, three buffered solutions were prepared: A_DNA_, B_DNA_, and C_DNA_ (10 μM). These solutions were mixed to produce four solutions corresponding to the input DNA patterns. The solutions were stored overnight prior to experiments to ensure complete hybridization. In experiments using a four-droplet system, A_DNA_, B_DNA_, and C_DNA_ (100 μM) were injected into each droplet to produce concentrations of 10 μM.

### DNA design and free energy calculation

To confirm that the DNA hybridized as intended, thermodynamic analysis was performed for each input case using NUPACK (California Institute of Technology) [20]. This analysis was performed at 25°C using a DNA concentration of 10 μM in 1.0 M KCl buffered electrolyte solution. Hybridization occurred and produced the intended secondary structures. The free energy of the secondary structures was calculated as shown in Table 2.

### Data recording and analysis

All measurements were performed at 23 ± 1°C in a clean room with less than 60% humidity inside a Faraday cage. The channel currents were amplified and recorded with a JET path-clamp amplifier (Tecella, Costa Mesa, CA, USA). The data were filtered at 1 kHz at a sampling rate of 5 kHz, and then analyzed using Clampfit (Axon Instruments, USA). DNA translocation and blocking were detected when >80% of open μHL channel currents were inhibited. The current-blocking events were counted by differentiating blocking events with a duration ≤ 1 s from those > 1 s.

### Device fabrication

An image of the device is shown in Fig 4a. A poly (methyl methacrylate) (PMMA) substrate (Mitsubishi Rayon, Tokyo, Japan) was used as the material for the device. The device was fabricated as follows. Four wells and grooves between wells were manufactured on a PMMA plate via micro-machining (MM-100, Modia Systems, Saitama, Japan). Each well contained a through-hole in the bottom. All four circular wells had a diameter of 4.0 mm and a depth of 2.0 mm. A polymeric film made of parylene (polychloro-p-xylylene) with a thickness of 5 μm was patterned to contain five pores (150 μm in diameter), and then sandwiched between 200-μm-thick PMMA films using adhesion bonding (Super X, Cemedine Co., Ltd, Tokyo, Japan). The PMMA films, including the polymeric film, were inserted into the groove using the same adhesive bond to separate the wells. The holes were filled with Ag/AgCl paste, enabling control of the applied voltages (Fig 4b). Cr/Ag films were vapor-deposited from the back side and patterned on the PMMA plate as electrical connections between the wells and a patch-clamp amplifier for electrical recording. This wiring was covered with another PMMA plate bonded to the first PMMA plate by thermocompression bonding for 20 min at 120°C with an applied force of approximately 5 × 10^5^ N m^−2^.

### Bilayer lipid membrane preparation

We used the droplet contact method (DCM) for rapid, simple lipid bilayer formation. Lipid bilayers were formed as follows. The wells of the device were filled with *n*-decane (3 μL) containing phospholipids (EggPC (20 mg mL^-1^) for the double-well chips and EggPC (14 mg mL^-1^) and cholesterol (7 mg mL^-1^) for the four-well chips). Droplets of buffer solution (16 μL) were dispensed into each of the wells by pipetting, producing lipid monolayers that self-assembled on the surfaces of the aqueous droplets. When two droplets come into contact, a BLM autonomously forms at the interface of the two adjoining lipid monolayers in the micropores of the hydrophobic film after several minutes. The thin film prevents the two droplets from merging. In addition, it has been reported that reducing the BLM formation area can decrease electrical noise and increase BLM stability [15]. Furthermore, a network of lipid-coated droplets was formed by repeating the DCM. The geometry of the droplet network was such that four droplets were positioned at the corners and the center of an equilateral triangle.

## Supporting Information

S1 Fig A representative result of gel electrophoresis. A_DNA_, B_DNA_, C_DNA_, A_DNA_+C_DNA_, B_DNA_+C_DNA_, and A_DNA_+B_DNA_+C_DNA_ indicate the DNA samples in each lane. Double-stranded DNA molecules composed of A_DNA_+B_DNA_+C_DNA_ produced a main band at the same location as the double-stranded 40-bp reference DNA. A_DNA_+C_DNA_ and B_DNA_+C_DNA_ produced a main band below the band for A_DNA_+B_DNA_+C_DNA_ above 20-bp reference. It was indicated that these DNA samples had lower molecular weight than the A_DNA_+B_DNA_+C_DNA_. The bands appered above 20-bp reference due to their single-stranded region. C_DNA_ produced a band below the other DNAs. No bands were observed in the A_DNA_ and B_DNA_ lanes. The results indicate that DNA hybridization occurred as planned.
(TIF)

S2 Fig Bar graph of output for 10 current inhibitions immediately after αHL reconstitution for each input. The obtained current inhibitions were differenciated and counted into two groups, (a) greater than 1 s and (b) not greater than 1 s. The calculated difference of a-b are shown as bar graph and the corresponding outputs accoding to the computing protocol are also shown. The standard errors were obtained based on three operations.
(TIF)

S3 Fig Construction procedure of a four-droplet network. (a) Injection of aqueous buffer solutions into each well, which were filled with oil, dispersing EggPC and Cholesterol. SLO monomers were contained in the input droplets and αHL monomers were contained in the operation droplet. (b) BLM formations at the interface of the droplets. (c) The SLO and αHL reconstitution to form nanopores into the BLMs. (d) Injections of input DNA strands and complementary DNA to the input droplets and the operation droplet.
(TIF)

S4 Fig Experiment to quantify the relationship between DNA concentration and translocation rate of Poly T50 DNA. (a) Schematic image of DNA translocation experiment. 16 μL of droplets were used where the consentration of αHL was 30 nM. Applied voltage was 120 mV to translocate the contained ssDNA. (b) Experimental results of the relationship between DNA concentration and translocation rate. The standard deviations were obtained based on the results of three experiments.
(TIF)

S1 Scheme Computing protocol for NAND operation. (TIF)

S1 Text Additional information on experiments. (DOCX)
