# Supplementary figures and images for: Logic Gate Operation by DNA Translocation through Biological Nanopores
Source: PLoS One. 2016 Feb 18;11(2):e0149667. doi: 10.1371/journal.pone.0149667 (PMC4758725; doi:10.1371/journal.pone.0149667)

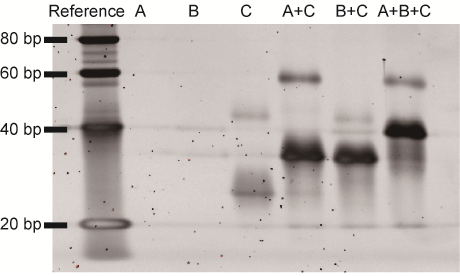

Supplement: S1 Fig — ADNA, BDNA, CDNA, ADNA+CDNA, BDNA+CDNA, and ADNA+BDNA+CDNA indicate the DNA samples in each lane. Double-stranded DNA molecules composed of ADNA+BDNA+CDNA produced a main band at the same location as the double-stranded 40-bp reference DNA. ADNA+CDNA and BDNA+CDNA produced a main band below the band for ADNA+BDNA+CDNA above 20-bp reference. It was indicated that these DNA samples had lower molecular weight than the ADNA+BDNA+CDNA. The bands appered above 20-bp reference due to their single-stranded region. CDNA produced a band below the other DNAs. No bands were observed in the ADNA and BDNA lanes. The results indicate that DNA hybridization occurred as planned. (TIF) [file pone.0149667.s001.tif]

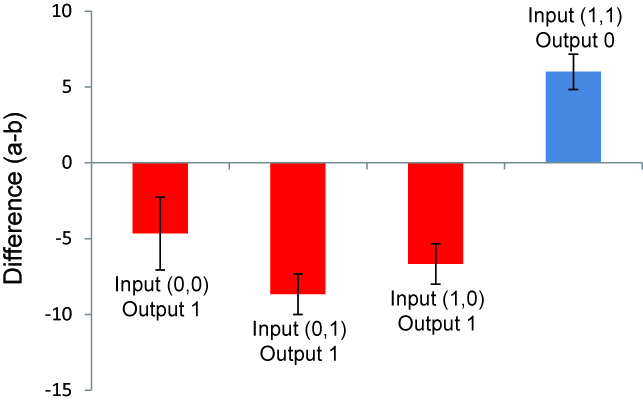

Supplement: S2 Fig — The obtained current inhibitions were differenciated and counted into two groups, (a) greater than 1 s and (b) not greater than 1 s. The calculated difference of a-b are shown as bar graph and the corresponding outputs accoding to the computing protocol are also shown. The standard errors were obtained based on three operations. (TIF) [file pone.0149667.s002.tif]

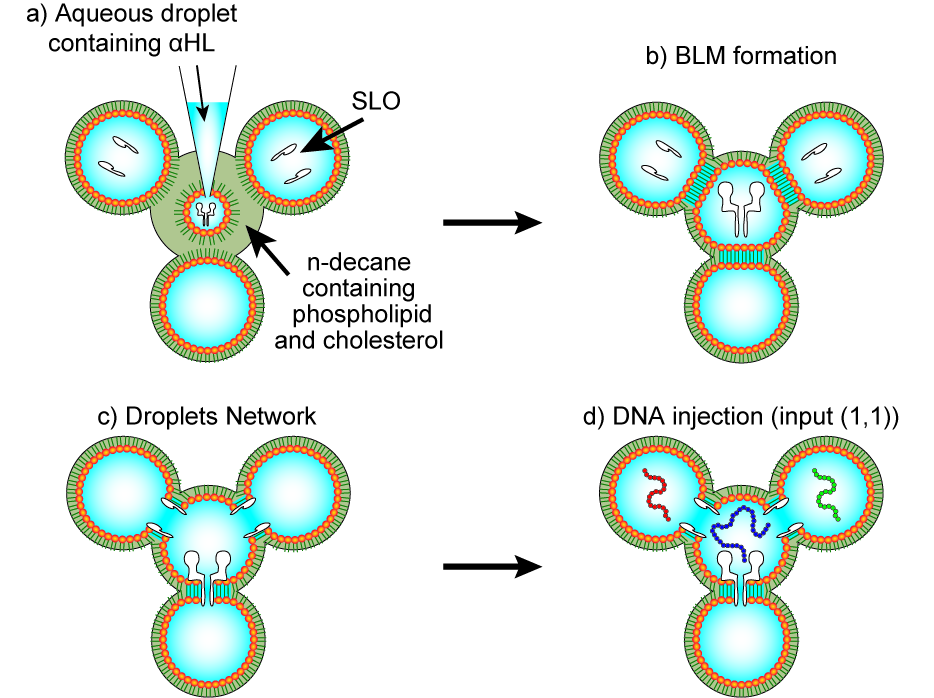

Supplement: S3 Fig — (a) Injection of aqueous buffer solutions into each well, which were filled with oil, dispersing EggPC and Cholesterol. SLO monomers were contained in the input droplets and αHL monomers were contained in the operation droplet. (b) BLM formations at the interface of the droplets. (c) The SLO and αHL reconstitution to form nanopores into the BLMs. (d) Injections of input DNA strands and complementary DNA to the input droplets and the operation droplet. (TIF) [file pone.0149667.s003.tif]

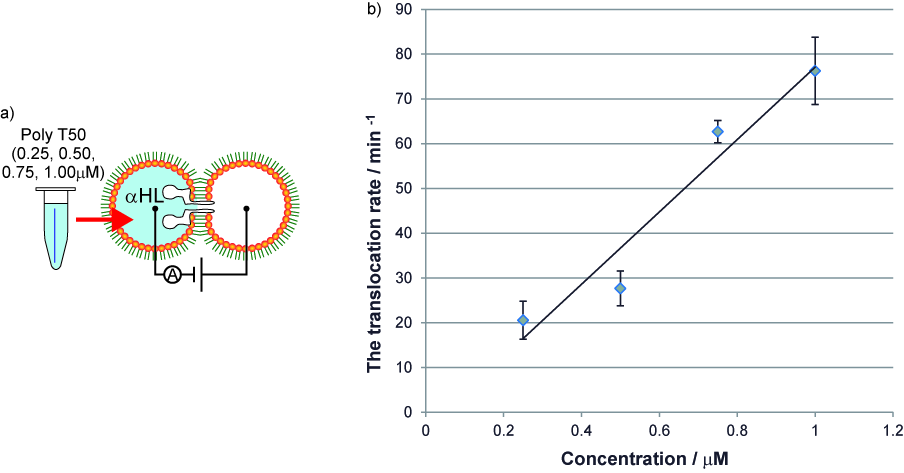

Supplement: S4 Fig — (a) Schematic image of DNA translocation experiment. 16 μL of droplets were used where the consentration of αHL was 30 nM. Applied voltage was 120 mV to translocate the contained ssDNA. (b) Experimental results of the relationship between DNA concentration and translocation rate. The standard deviations were obtained based on the results of three experiments. (TIF) [file pone.0149667.s004.tif]

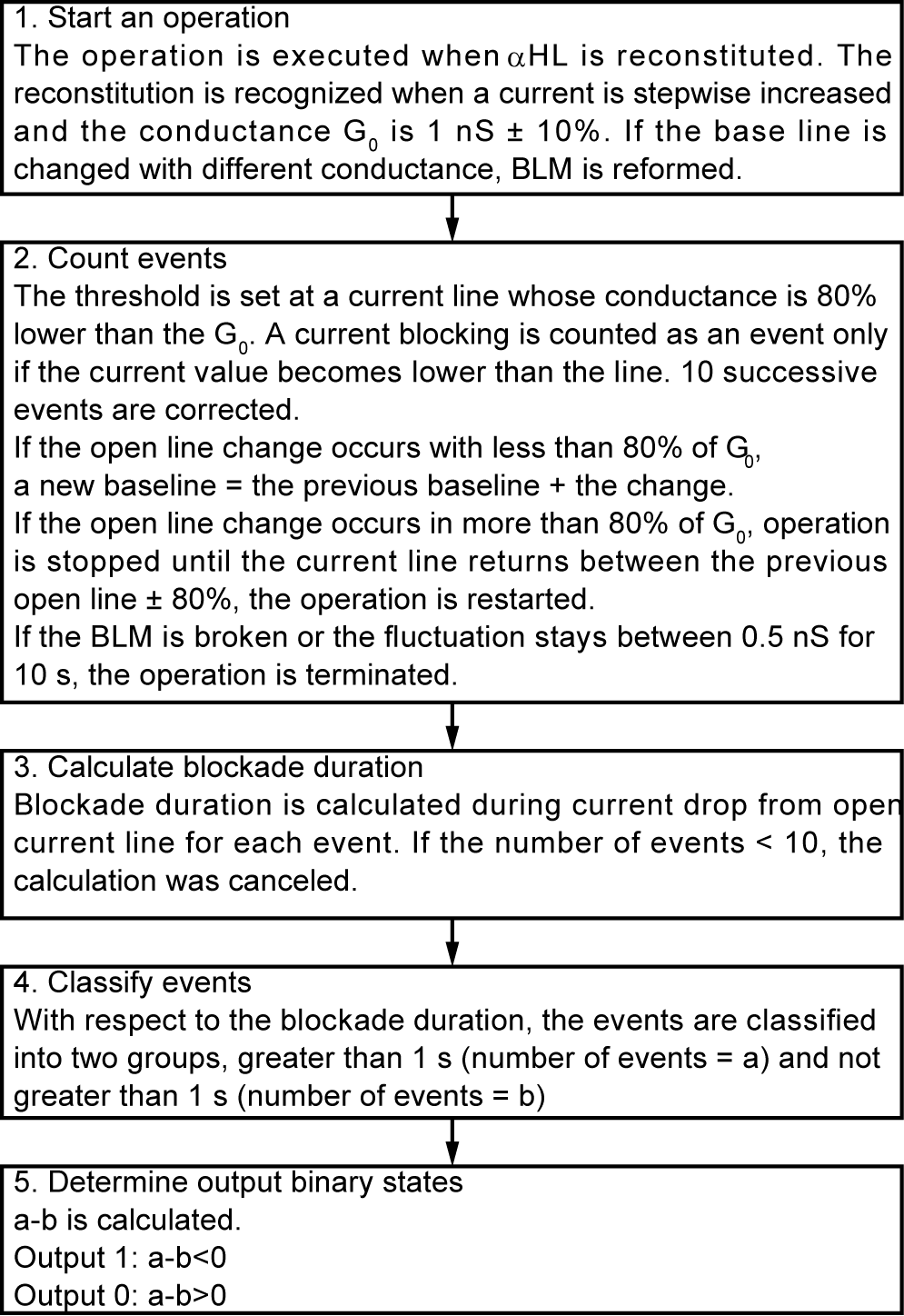

Supplement: S1 Scheme — (TIF) [file pone.0149667.s005.tif]
