# Supplementary material for: Logic Gate Operation by DNA Translocation through Biological Nanopores
Source: PLoS One. 2016 Feb 18;11(2):e0149667. doi: 10.1371/journal.pone.0149667 (PMC4758725; doi:10.1371/journal.pone.0149667)
Supplement: S1 Text — (DOCX) [file pone.0149667.s006.docx]

S1 Text. Additional information on experiments

**Gel Electrophoresis**

The intended DNA hybrization was confirmed by gel electrophoresis. In addition to the input patterns, A_DNA_, B_DNA_, and C_DNA_ alone were mixed with 10× loading buffer (Takara Bio Inc., Shiga, Japan) and prepared for electrophoresis. A 15% acrylamide gel (ATTO Corp, Tokyo, Japan) subjected to electrophoresis in a Tris-borate-EDTA buffer (pH8.3 - 8.5; 0.089 M Tris; 0.089 M boric acid, 2.6 mM EDTA: Takara Bio Inc., Shiga, Japan). DNA samples were electrophoresed at 150 V for 90 min with reference (20-bp DNA Ladder (Dye Plus): Takara Bio Inc., Shiga, Japan). The gel was visualized using SYBR gold (Thermo Fisher Scientific Inc., Waltham, MA, USA); gels were immersed into a solution containing SYBR gold for 15 min. S1 Fig shows the result of the gel electrophoresis.

**Verification of DNA translocation**

**αHL**

We conducted an experiment to quantify ssDNA translocation through the nanopore. It has previously reported that the ssDNA translocation rate through αHL is proportional to the ssDNA concentration.^[1]^ For various concentrations (0.25, 0.50, 0.75, and 1.00 μM) of poly T50 ssDNA, translocation rates were experimentally examined using DWC (S4 Fig). As shown in S4 Fig b, the proportionality between DNA concentration and translocation rate was verified. A rate constant of the translocation was calculated as *K* (=76.1 μM^−1^min^−1^). To obtain the amount of translocated DNA through αHL over time, the following equation was used:

| $-\text{VN}_{\text{A}}\frac{\text{dC}_{\text{cis}}}{\text{dt}}\text{=KC}_{\text{cis}}\text{N}_{\text{H}}$ | (1) |
| --- | --- |

where *V* is the volume of droplets, *N_A_* is Avogadro's number, *C_cis_* is the concentration of ssDNA strands in the droplet of the DNA source, and *N_H_* is the number of reconstituted αHL. Since the value of *K* was extremely small, the concentration in a droplet after DNA translocation did not reach a measurable value in a realistic time frame.

**SLO**

Streptolysin O (SLO) was selected because SLO monomers assemble to form a pore with a diameter of up to approximately 25 nm, which is larger than that of αHL.^[2-4]^ Owing to its large diameter, the SLO pore is expected to allow simultaneous ssDNA translocations. Real-time observation of single ssDNA translocation was not possible because the size of ssDNA is too small to detect electrically in the SLO channel recordings. To confirm the ssDNA translocation through the nanopore of SLO, we conducted an experiment. The solution of SLO and ssDNA with 20-mers was prepared and injected into the input droplet (grounded well) of the DWC. Several minutes after applying voltage across the two droplets, a 1.5 μl of the solution in the output droplet was extracted and its concentration was measured by inspecting the UV absorbance with NanoDrop 2000 (Thermo Fisher Scientific Inc., Waltham, MA, USA). The maintenance of a SLO pore formation with 20 – 30 nm in diameter was observed with the channel current recording. In the experiment, 25 minutes after starting ssDNA translocation, the concentration reached 2.0 μM, which was 20% of the original source concentration. The experimental results indicated the concentration of ssDNA could reach a measurable value for several minutes with large number of SLO pores.

**References**

1. Henrickson SE, Misakian M, Robertson B, Kasianowicz JJ. Driven DNA transport into an asymmetric nanometer-scale pore. Phys Rev Lett. 2000;85: 3057–60. doi:10.1103/PhysRevLett.85.3057

2. Sekiya K, Akagi T, Tatsuta K, Sakakura E, Hashikawa T, Abe A, et al. Ultrastructural analysis of the membrane insertion of domain 3 of streptolysin O. Microbes Infect. 2007;9: 1341–50. doi:10.1016/j.micinf.2007.06.010

3. Sekiya K, Danbara H, Yase K, Futaesaku Y. Electron microscopic evaluation of a two-step theory of pore formation by streptolysin O. J Bacteriol. 1996;178: 6998–7002. Available: http://link.aps.org/doi/10.1103/PhysRevE.75.021906

4. Zitzer A, Westover EJ, Covey DF, Palmer M. Differential interaction of the two cholesterol-dependent, membrane-damaging toxins, streptolysin O and Vibrio cholerae cytolysin, with enantiomeric cholesterol. FEBS Lett. 2003;553: 229–31. doi:10.1016/S0014-5793(03)01023-8
